# **Study of thermo-regulation as a worsening marker of experimental sepsis in an animal model** *

**DOI:** 10.1590/1518-8345.3364.3290

**Published:** 2020-09-07

**Authors:** André Luiz Thomaz de Souza, Marcelo Eduardo Batalhão, Evelin Capellari Cárnio

**Affiliations:** 1 Universidade de São Paulo, Escola de Enfermagem de Ribeirão Preto, PAHO/WHO Collaborating Centre at the Nursing Research Development, Ribeirão Preto, SP, Brazil.; 2 Faculdades Integradas do Vale do Ribeira, Faculdade de Enfermagem, Registro, SP, Brazil.

**Keywords:** Endotoxemia, Sepsis, Body Temperature, Nitric Oxide, Serum Lactate, Biomarkers, Endotoxemia, Sepse, Temperatura Corporal, Óxido Nítrico, Lactato Sérico, Biomarcadores, Endotoxemia, Sepsis, Temperatura Corporal, Óxido Nítrico, Lactato Sérico, Biomarcadores

## Abstract

**Objective:**

to analyze variations in body temperature and in plasma nitrate and lactate concentrations in rats submitted to the experimental sepsis model.

**Method:**

a total of 40 rats divided equally into five groups. The induction of endotoxemia was performed with intravenous administration of lipopolysaccharide, 0.5 mg/Kg, 1.5 mg/Kg, 3.0 mg/Kg, and 10 mg/Kg, respectively. The control group received 0.5 mL of saline solution. The experiment lasted six hours, with evaluations performed at 0 (baseline data), 2^nd^, 4^th^, and 6^th^hours.

**Results:**

The animals that received doses up to 3.0 mg/kg showed a significant increase in body temperature compared to the group with 10 mg/kg, which showed a decrease in these values. The increase in plasma nitrate and lactate concentrations in the groups with lipopolysaccharide was significantly higher than in the group that received the saline solution and was correlated with the increase in body temperature.

**Conclusion:**

the variations in body temperature observed in this study showed the dose-dependent effect of lipopolysaccharide and were correlated with the increase in the concentrations of nitrate and plasma lactate biomarkers. The implications of this study are the importance of monitoring body temperature, together with the assessment of these pathophysiological markers, which suggest worsening in the prognosis of sepsis.

## Abstract (pt)

**Objetivo:**

analisar as variações na temperatura corporal e nas concentrações de nitrato e lactato plasmáticos em ratos submetidos ao modelo de sepse experimental.

**Método:**

foram utilizados 40 ratos divididos igualmente em cinco grupos. A indução da endotoxemia foi realizada com administração endovenosa de lipopolissacarídeo, respectivamente 0,5 mg/Kg, 1,5 mg/Kg, 3,0 mg/Kg e 10 mg/Kg. O grupo controle recebeu 0,5 mL de solução salina. O experimento teve duração de seis horas, com avaliações realizadas na 0 (dados basais), 2^a^, 4^a^ e 6^a^ hora.

**Resultados:**

os animais que receberam doses de até 3,0 mg/Kg apresentaram aumento significativo na temperatura corporal em relação ao grupo com 10 mg/Kg, que apresentou diminuição nesses valores. O aumento nas concentrações de nitrato e lactato plasmáticos nos grupos com lipopolissacarídeo foi significativamente superior ao grupo que recebeu salina e esteve correlacionado com o aumento na temperatura corporal.

**Conclusão:**

as variações na temperatura corporal observadas neste estudo mostraram efeito dose dependentes de lipopolissacarídeo e estiveram correlacionadas com o aumento nas concentrações dos biomarcadores nitrato e lactato plasmáticos. O estudo traz como implicações, a importância no monitoramento da temperatura corporal, em conjunto com a avaliação destes marcadores fisiopatológicos, os quais sugerem agravamento no prognóstico da sepse.

## Abstract (es)

**Objetivo:**

analizar las variaciones de la temperatura corporal y de las concentraciones de nitrato y lactato en plasma en ratones sometidos a un modelo de sepsis experimental.

**Método:**

se utilizaron 40 ratones divididos en cinco grupos iguales. La inducción de la endotoxemia se realizó mediante administración intravenosa de 0,5 mg/Kg, 1,5 mg/Kg, 3,0 mg/Kg y 10 mg/Kg de lipopolisacárido, respectivamente. El grupo de control recibió 0,5 mL de solución salina. El experimento duró seis horas, con evaluaciones realizadas a la hora 0 (datos de referencia) y a la 2^a^, 4^a^ y 6^a^hora.

**Resultados:**

los animales que recibieron dosis de hasta 3,0 mg/kg presentaron un aumento significativo de la temperatura corporal, en comparación con el grupo al que se le administró 10 mg/kg, que presentó una disminución de dichos valores. En los grupos a los que se les administró lipopolisacárido, el aumento en las concentraciones de nitrato y lactato en plasma fue significativamente mayor que en el grupo al que se le administró la solución salina y estuvo correlacionado con el aumento de la temperatura corporal.

**Conclusión:**

las variaciones de la temperatura corporal observadas en este estudio mostraron que los efectos dependieron de la dosis de lipopolisacárido, y estuvieron correlacionadas con el aumento en la concentración de biomarcadores, como el nitrato y lactato en plasma. El estudio reveló la importancia del control de la temperatura corporal, junto con la evaluación de estos marcadores fisiopatológicos, que sugieren un empeoramiento en el pronóstico de la sepsis.

## Introduction

Despite the large number of studies available in the literature, limitations are still found in the understanding of the pathophysiological mechanisms, which result in high rates of sepsis-related morbidity and mortality in Intensive Care Units (ICUs)^(^ 1 ^)^. The clinical course of the disease can lead to a worsening of the prognosis, when changes occur to the stages of severe sepsis and septic shock^(^ 2 ^)^. This change represents a mortality rate ranging from 10% to 40%^(^ 3 ^-^ 4 ^)^.

Among the clinical manifestations presented in the disease, body temperature is an important cardinal sign about the health conditions, whose strict control of thermo-regulation can increase the chances of survival of the patients^(^ 5 ^)^. However, the mechanisms that result in an ineffective thermo-regulation during the most severe stages of sepsis, mainly related to hypothermia, remain misunderstood^(^ 1 ^,^ 5 ^)^.

The exacerbated inflammatory response and infection are determining factors in the clinical evolution of sepsis^(^ 6 ^)^, which accompanies the increase in the production of pro-inflammatory cytokines (interleukin - (IL-) 1β, tumor necrosis factor - (TNF-) α, and interferon - (IFN-) γ) or of anti-inflammatory cytokines (interleukin - (IL-) 10, and transforming growth factor - (TGF-) β)^(^ 7 ^-^ 8 ^)^. During the immune response, an increase in the production of reactive oxygen species is also observed, for example, Nitric Oxide (NO)^(^ 9 ^)^.

NO formation occurs endogenously from L-arginine catabolism, resulting in the formation of L-citrulline and NO through enzymatic reaction of the NO synthase (NOS) enzyme^(^ 10 ^-^ 11 ^)^. Among the NOS isoforms produced in the body, inducible NO (iNOS) participates in the immune response and can be produced through external stimuli, such as in the presence of lipopolysaccharide (LPS) and pro-inflammatory cytokines^(^ 10 ^-^ 11 ^)^.

In addition to the increase in NO during the stages of sepsis, plasma lactate can also be found in high concentrations. This increase can be considered a marker of tissue hypoperfusion when found in concentrations ˃1.0 mmol/L^(^ 2 ^)^. The elevation of plasma lactate results from the production of energy by anaerobic glycolytic^(^ 12 ^)^, mainly observed in septic shock. Although these two biomarkers show a significant increase in the course of the disease, only lactate is used as a predictor of severity in the clinical practice.

Thermo-regulation has been extensively investigated in experimental models of sepsis and septic shock^(^ 13 ^-^ 14 ^)^, showing that the same inflammatory agent can induce both fever and hypothermia^(^ 14 ^)^. However, the mediators that participate in hypothermia are still misunderstood^(^ 15 ^)^. It is believed that NO can influence the control of body temperature.

A number of studies in animals have shown different effects of NO on temperature, whether in situations where donors or inhibitors of its synthesis are administered^(^ 16 ^-^ 17 ^)^. In a study with an animal model submitted to endotoxemia (a condition similar to sepsis), it was identified that NO acts as a pyretic mediator of fever. The study showed that the pharmacological administration of NO synthesis inhibitors resulted in a decrease in body temperature during the febrile response^(^ 16 ^)^. In contrast, the reduction of febrile states was also observed when administering NO donors in the lateral cerebral ventricle of rabbits, revealing a stimulus in the antipyretic activity in the central nervous system^(^ 17 ^)^.

A number of studies involving the measurement of NO production during sepsis in humans are rare; however, in general, they evidence a small increase in this production^(^ 18 ^)^. It is suggested that this increase may be correlated with the decrease in body temperature in patients with septic shock^(^ 19 ^)^.

With regard to lactate, as well as NO, the relation between the concentrations of this pathophysiological marker and body temperature in sepsis is little discussed in the literature. In the clinical context, high lactate concentrations serve as a global parameter to identify metabolic impairment in critically ill patients^(^ 12 ^,^ 20 ^)^.

During the nursing practice, body temperature control is used as a reference of the patient’s pathophysiological conditions. The increase or decrease in temperature signals situations that require immediate interventions, with a focus on preserving homeostasis. Therefore, the monitoring of the vital signs allows the nursing team to early identify organic changes suggestive of sepsis and/or other complications^(^ 21 ^)^. In this scenario, the management of health care performed by nurses requires knowledge about the morphofunctional changes evidenced by the organism.

Faced with the problem involving the stages of sepsis and its clinical manifestations, this study aimed to analyze variations in body temperature and plasma nitrate and lactate concentrations in rats submitted to the experimental sepsis model. This research is important to expand the understanding about the participation of biomarkers in thermo-regulation.

## Method

An experimental study carried out with 40 Wistar rats, aged 8 to 12 weeks old and with a body mass between 200 and 300 grams. The animals were kept in ventilated shelves with controlled room temperature (25°C±2°C). In addition, they were exposed to a 12/12 hour light-dark cycle and had free access to water and a balanced commercial diet. To avoid circadian variations, all the experiments were always started between 8:00 am and 10:00 am. The experimental stages were carried out in accordance with the Ethics Commission on Animal Use of the Ribeirão Preto Nursing School at the University of São Paulo - USP; Ribeirão Preto, SP, Brazil (protocol No. 15.737.22.0).

The experimental protocol adopted in this study involved the intravenous administration of 0.9% physiological solution (saline) or different concentrations of LPS from Serotype 0111:B4 *Escherichia coli* (Sigma-Aldrich^®^, St. Louis, MO, USA), the body temperature records (°C), and blood collection for plasma nitrate (µM) and lactate (mmol/L) analysis. The 40 animals were equally distributed in five groups with eight animals each. One group (control) received 0.5 mL of saline solution and the others received doses of LPS equal to 0.5 mg/kg, 1.5 mg/kg, 3.0 mg/kg, and 10 mg/kg, respectively.

For the control of body temperature, six days before the induction of the experimental models, a capsule of *datalogger* was inserted into the peritoneal cavity, through a surgical incision in the abdominal wall, under general anesthesia with 2% xylazine hydrochloride (2 mg/mL) and 10% ketamine hydrochloride (10 mg/mL), administered in a single dose of 0.10 mL for each 100 g of animal weight, intraperitoneally (IP).

The *dataloggers* capsules were previously programmed to record body temperature (°C) at 15-minute intervals for 24-hour periods. After insertion, the incision site was sutured with resorbable threads. At the end of the surgery, the animals received prophylaxis with benzylpenicillin (120,000 IU) and streptomycin (50 mg), as well as analgesia with flunixinameglumine (2.5 mg/kg) intramuscularly (IM) in a single dose. The post-surgical recovery time corresponded to five days.

For the administration of LPS or saline solution intravenously, the rats were again anesthetized and had the jugular vein cannulated according to the technique described in the literature^(^ 22 ^)^. Heparinized silastic cannulas (Sigma-Aldrich^®^) were used, with a total length of 10 cm. Approximately 1.7 cm were inserted into the jugular vein towards the right atrium. The other parts of the cannula were positioned on the animal’s back with the help of a trocar and fixed with cotton threads in blocks with simple stitches.

Immediately after cannulation of the jugular vein, the animals had the femoral artery cannulated. A polyethylene (PE) cannula, consisting of a 4.5 cm long PE-10 segment, connected to a 15 cm PE-50 catheter was inserted into the femoral artery towards the abdominal aorta. At the end, the cannula was fixed in place and its free end was exteriorized, and also fixed on the animal’s back. This cannula was used to collect blood samples.

After the vessel cannulation procedures were completed, the animals again received the same prophylaxis performed after the insertion of the *datalogger* and were housed in the ventilated shelf, remaining with free access to water and until the induction of the experimental models that occurred after 24 hours.

In the experiment room, the room temperature was maintained at 25±2°C. Initially, the animals were subjected to adaptation in this location so that the thermal balance of the body with room temperature would occur. After the six hours of experiment, the animals were sacrificed and the *datalogger* capsule was removed from the peritoneal cavity. Both the programming and acquisition of the temperature records were performed using the *SubCue Analyzer* software.

In order to evaluate plasma nitrate and lactate concentrations, blood samples were collected using the femoral cannula (0.4 mL) at 0 h (baseline data), 2 h, 4 h, and 6 h after the induction of the experimental models. After lactate measurement, the blood samples were stored in polypropylene tubes containing sodium heparin (1,500 IU/tube), and were immediately placed in ice. Volume replacement, referring to the blood aliquots taken from the animals at the times described, was performed in the same proportion (0.4 mL) with 0.9% physiological solution.

The determination of plasma lactate was performed by the ACCUTREND PLUS (Roche^®^) portable device, using specific reagent strips (Accusport BM - Lactate). Immediately after blood collection, a small aliquot was placed on the reagent strip and the remaining parts stored at -20°C, for later measurement of plasma nitrate. With the strip filled with blood, the device allowed identifying a range of values from 0.8 to 22 mmol/L, with a measurement time of 60 seconds.

Plasma nitrate was determined through the Sievers system (Instruments Nitric Oxide Analyzer). After centrifuging the blood at 5,000 rpm for 10 minutes, the plasma samples obtained were deproteinated using cold absolute ethanol. Subsequently, these samples were injected into a container with vanadium trichloride (VCI_3_), which converts nitrate to NO. The NO produced was detected by ozone induced by chemiluminescence. The peak NO concentrations of these samples were determined using the standard curve, established with sodium nitrate solutions of various concentrations (0; 7.5; 15; 30; 60; 120; and 240 µM).

In the data analysis, One-Way ANOVA was used, followed by the Tukey post-test to test the differences between the means of the experimental groups at 0, 2, 4, and 6 hours after the administration of LPS or of saline. The mean values obtained at 0 hours were considered as baseline values. In addition, the Pearson Correlation Test and/or the *Spearman* correlation test were used to identify possible associations among the investigated variables and according to the LPS dose which was administered. The results were presented in graphs of mean and Standard Error of the Mean (SEM). The level of significance adopted for all the tests was 0.05 (5%).

## Results

**Figure 1:**
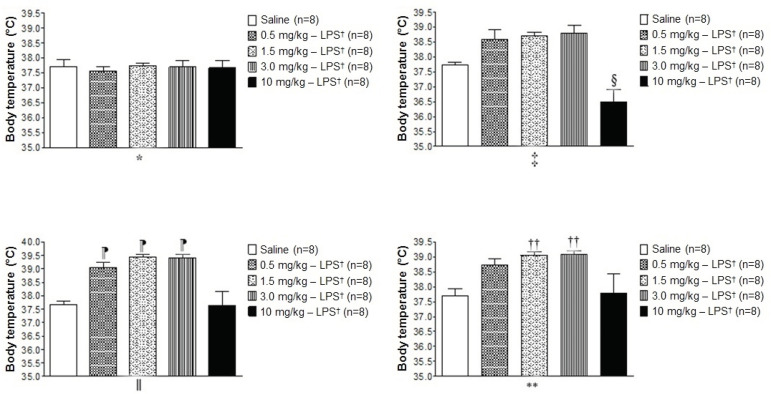
Effect of the administration of different doses of LPS on body temperature

The administration of LPS at a concentration of 10 mg/kg significantly reduced the animals’ body temperature when compared to the other groups after two hours of experiment (Figure 1 ^‡^). At the fourth hour, the temperature of the animals with 0.5, 1.5, and 3.0 mg/kg of LPS was higher when compared to the saline group and 10 mg/kg, the values being statistically significant (Figure 1 ^ǁ^). At the sixth hour, in the groups with LPS there were no statistical differences. However, the body temperature of the groups with 1.5 and 3.0 mg/kg of LPS remained statistically higher than the saline group (Figure 1**).

*0 hour - measured before the administration of LPS or saline; ^†^LPS = Lipopolysaccharide; ^‡^2 hours later; ^§^Indicates a statistical difference between the groups with 10 mg/Kg - LPS *versus* the groups with 0.5, 1.5, and 3.0 mg/Kg - LPS; ^‖^4 hours later; ^¶^Indicates a statistical difference between the groups with 0.5, 1.5, and 3.0 mg/Kg - LPS *versus* the saline group and the group with 10 mg/kg - LPS; **6 hours later; ^††^Indicates a statistical difference between the groups with 1.5 and 3.0 mg/Kg - LPS *versus* the saline group. A statistical difference was identified by One-Way ANOVA followed by Tukey’s multiple comparison post-test (p˂0.05)

**Figure 2:**
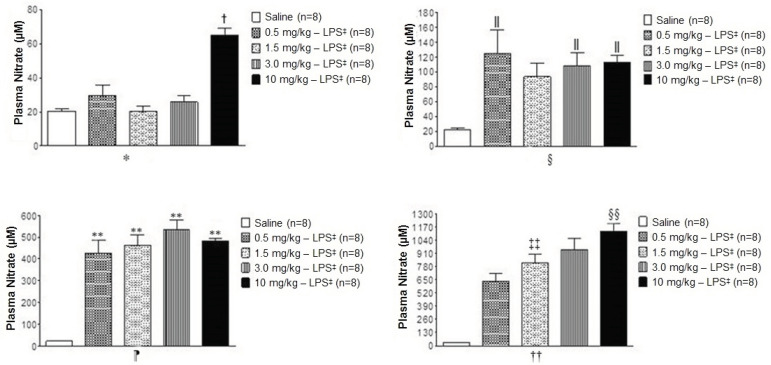
Effect of administration of different doses of LPS on plasma nitrate concentration

The plasma nitrate concentrations were statistically different in the animals with LPS when compared to the saline group (Figure 2). At zero hours (Figure 2*), the animals with 10 mg/kg of LPS showed higher means than the other groups; however, the plasma nitrate concentrations did not exceed 100 µM, different from the levels observed at the other hours. Two hours after the administration of LPS, significant differences were observed between groups with 0.5, 3.0, and 10 mg/kg - LPS when compared to the saline group (Figure 2 ^§^). At the fourth hour, the groups with LPS differed statistically from the saline group, with a significant increase in nitrate concentrations, reaching values over 500 µM (Figure 2 ^¶^). Six hours after the administration of LPS, the significant difference with the saline group remained; in addition, the groups with higher doses of LPS showed statistically higher concentrations of plasma nitrate reaching values over 900 µM (Figure 2 ^††^).

*0 hour - measured before the administration of LPS or saline; ^†^Indicates a statistical difference between the group with 10 mg/kg - LPS *versus* the saline groups, 0.5, 1.5, and 3.0 mg/Kg - LPS; - ^‡^LPS = Lipopolysaccharide; ^§^2 hours later; ^**‖**^ Indicates a statistical difference between the groups with 0.5, 3.0, and 10 mg/Kg - LPS *versus* the saline groups and the group with 1.5 mg/kg - LPS; ^¶^4 hours later; **Indicates a statistical difference between the groups with 0.5, 1.5, 3.0, and 10 mg/Kg - LPS *versus* the saline group; ^††^6 hours later; ^‡‡^Indicates a statistical difference between the group with 1.5 mg/Kg - LP *versus* the group with 3.0 mg/Kg - LPS; ^§§^Indicates a statistical difference between the group with 10 mg/Kg - LPS *versus* the groups with 0.5 and 1.5 mg/Kg - LPS. A statistical difference was identified by One-Way ANOVA followed by Tukey’s multiple comparison post-test (p˂0.05)

**Figure 3:**
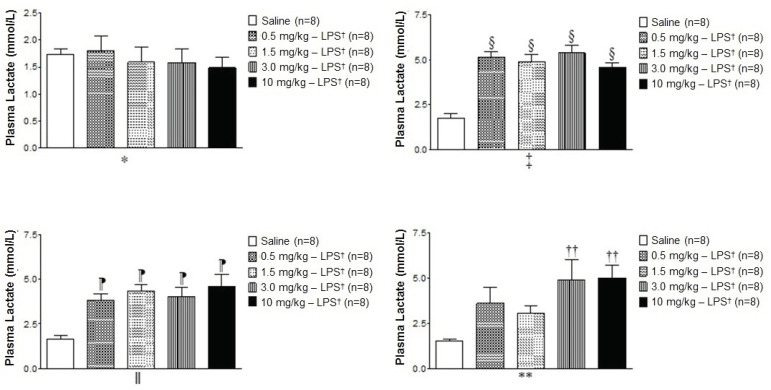
Effect of the administration of different doses of LPS on plasma lactate concentrations

The plasma lactate concentrations at zero hours did not differ between the experimental groups; however, at the second and fourth hours there were significant increases in these concentrations leading to a statistically significant increase when comparing LPS *versus* saline (Figures 3 ^‡^and 3^ǁ^ ). After six hours, significant differences were observed only when comparing the groups with higher doses of LPS, 3.0 and 10 mg/kg, respectively, when compared to the saline group (Figure 3**).

*0 hour - measured before the administration of LPS or saline; ^†^LPS = Lipopolysaccharide; ^‡^2 hours later; ^§^Indicates a statistical difference between the groups with 0.5, 1.5, 3.0, and 10 mg/Kg - LPS *versus* the saline group; ^‖^4 hours later; ^¶^Indicates a statistical difference between the groups with 0.5, 1.5, 3.0, and 10 mg/kg - LPS *versus* the saline group; **6 hours later; ^††^Indicates a statistical difference between the groups with 3.0 and 10 mg/kg - LP *versus* the saline group. A statistical difference was identified by One-Way ANOVA followed by Tukey’s multiple comparison post-test (p˂0.05)

**Figure 4:**
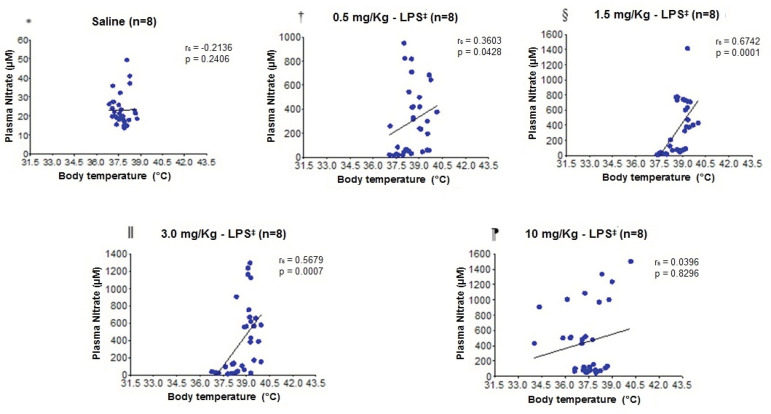
Dispersion diagram for correlation analysis between plasma nitrate concentrations and body temperature

In the correlation analysis between plasma nitrate concentrations and body temperature of the experimental models (Figure 4), no significant differences were found in the saline group and the group with 10 mg/Kg - LPS. However, there was a significant correlation in the 0.5 mg/kg - LPS, 1.5 mg/Kg - LPS, and 3.0 mg/Kg - LPS groups. The significant correlation shown in Figure 4 suggests that the higher the plasma nitrate concentration, the higher the body temperature.

*Saline; ^†^0.5 mg/Kg - LPS; ^‡^LPS = Lipopolysaccharide; ^§^1.5 mg/Kg - LPS; ‖3.0 mg/Kg - LPS; ^¶^10 mg/Kg - LPS

**Figure 5:**
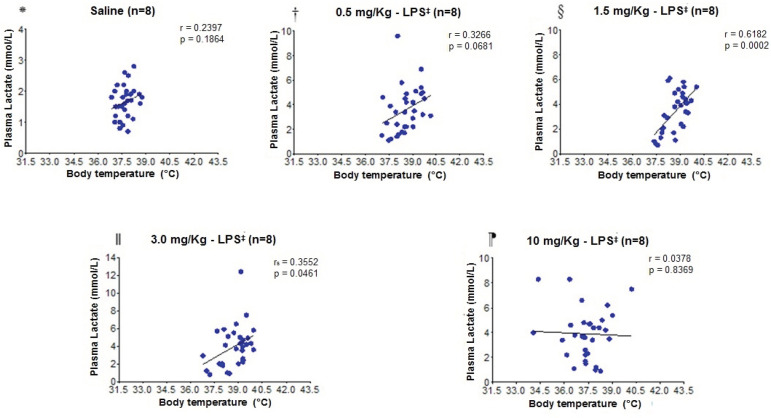
Dispersion diagram for correlation analysis between plasma lactate concentrations and body temperature

The correlation between the plasma lactate concentrations and the body temperature of the experimental models (Figure 5) did not present statistical differences in the saline, 0.5 mg/Kg - LPS, and 10 mg/Kg - LPS groups. However, the animals that received 1.5 mg/Kg - LPS and 3.0 mg/Kg - LPS showed a significant correlation, and the higher the plasma lactate concentration, the higher the body temperature value.

*Saline; ^†^0.5 mg/Kg - LPS; ^‡^LPS = Lipopolysaccharide; ^§^1.5 mg/Kg - LPS; ^‖^3.0 mg/Kg - LPS; ^¶^10 mg/Kg - LPS

## Discussion

This study shows, for the first time, the correlation between body temperature and mediators that participate in the pathophysiology of sepsis and septic shock (plasma NO and lactate) in animals submitted to experimental sepsis with different doses of LPS. The animals showed ineffective thermo-regulation, accompanied by an increase in the plasma concentrations of the analyzed physiological markers.

In humans, changes in body temperature identified in sepsis are also related to fever and/or hypothermia, which are characteristic signs for the screening and diagnosis of the disease^(^ 2 ^)^. It is suggested that fever is a frequent manifestation in sepsis. On the other hand, hypothermia is common in septic shock, being interpreted as a clinical worsening of the patient’s prognosis, increasing the chances of death^(^ 5 ^,^ 23 ^-^ 24 ^)^.

Although there are different attempts to reproduce sepsis and septic shock in animal models, it is important to interpret the results with caution, since rats have different responses than humans^(^ 24 ^)^. In cases of infection, humans usually have a fever and in some cases there may be a decrease in temperature, whereas rodents usually have a reduction in body temperature in the face of a significant infection^(^ 24 ^-^ 25 ^)^.

So far, the understanding of the participation of NO in the regulation of body temperature leads to different interpretations: some studies indicate that the inhibition of NO synthesis with the use of L-NAME (inhibitor of NO synthesis) injected into the peritoneum prevented fever in animals submitted to LPS administration^(^ 26 ^)^, suggesting that NO may act as a pyretic mediator of fever. On the other hand, it is also shown that the increase in NO concentrations in animals submitted to endotoxemia resulted in hypothermia^(^ 27 ^)^. In addition, the administration of NO donors in the intracerebroventricular region reduced fever in rabbits^(^ 17 ^)^, suggesting a central antipyretic effect.

In a study with humans carried out by researchers who work in our laboratory, a correlation was observed between the decrease in body temperature and the elevation of plasma NO concentrations in septic shock situations, which was not observed during sepsis^(^ 19 ^)^. However, we observed a positive correlation in the groups of animals that received lower doses of LPS, with body temperature values directly related to NO concentrations. This difference between the groups of animals that received different doses can be attributed to a greater resistance of experimental animals to LPS.

The correlation observed between NO doses and body temperature in endotoxemia, with lower doses of LPS, may suggest the action of NO, with its bactericidal property^(^ 28 ^)^, associated with increased body temperature, as a way of defending the body. Thus, the increase in temperature may be linked to greater activity of the immune system, with the production of prostaglandin E2 (PGE2) and consequently an increase in body temperature.

On the other hand, knowing the harmful effect of NO in high concentrations, the inverse correlation observed in a study with humans^(^ 19 ^)^ during septic shock can be indicative of failure in the body’s response capacity, associated with increased oxidative stress and consequently hypothermia.

Considering the different responses between humans and animals, the results of our study suggest that, probably, the higher concentration of NO in humans would result in a decrease in body temperature, since the opposite effect occurs in animals. This hypothesis has been investigated in studies with human beings, and the correlation between the increase in nitrate concentrations and the decrease in body temperature in septic shock has been confirmed^(^ 19 ^)^.

It should be noted that the intense decrease in body temperature observed in the group that received the highest dose of LPS (10 mg/kg), accompanied by the significant increase in nitrate concentrations, shows that the higher the dose administered, the lower the body temperature, confirming the notes found in the literature^(^ 29 ^)^.

The significant increase in plasma lactate concentrations in groups with LPS was also observed in our study. Both sepsis markers (nitrate and lactate), together with the assessment of the vital signs, are important indicators of the clinical severity of sepsis in humans. It should be noted that lactate is used as a parameter for the diagnosis of septic shock^(^ 30 ^)^. Since it is a mediator of difficult dosage in the clinical environment, the evaluation of NO is often restricted to scientific research.

In the stages of sepsis and in situations of endotoxemia, there is an increase in anaerobic metabolism and lactate production, which in turn alter the functioning of the immune cells^(^ 31 ^)^. The increase in this production may result in the negative regulation of glycolytic enzymes, specifically hexokinase and phosphofrutokinase, both in immune cells^(^ 32 ^)^ and in a variety of tissues^(^ 33 ^)^. Thus, considering the importance of aerobic glycolysis for the functioning of the immune cells in activity, the negative regulation of these enzymes under the influence of lactate implies the functional impairment of these cells^(^ 6 ^)^.

Recent studies have shown that decreased lactate production has resulted in improved animal survival^(^ 34 ^-^ 36 ^)^, while high lactate concentrations in peritoneal dialysis solutions inhibited LPS-induced maturation of dendritic cells (10 ng/mL)^(^ 37 ^)^. Lactate treatment also increased the production of genes associated with M2 (VEGF and Arg1) and markers (Fizz1, Mgl1, and Mgl2)^(^ 38 ^)^. M2 is an immuno-suppressive phenotype derived from the macrophages found in the late stages of sepsis; its increase may result in critical dysfunction in the immune system^(^ 38 ^)^.

In the adaptive immune system, the presence of high concentrations of lactate in the synovial fluid and in the joints of patients with rheumatoid arthritis, played a signaling role for the localization of T cells at the site of inflammation^(^ 32 ^)^. When carrying out *in vitro* experiments, the study authors point out that extracellular sodium lactate and lactic acid block the motility of CD4+ and CD8+ T cells, respectively^(^ 32 ^)^.

As in the animal models, the increase in lactate concentrations in sepsis and septic shock in humans is interpreted as a poor prognosis. This increase has an impact on the reduction of the survival chances^(^ 20 ^)^ and signals dysfunctions in the immune system^(^ 31 ^-^ 32 ^)^. In this context, our results reinforce the importance of monitoring lactate in experimental and clinical research studies, since it is an easy variable to measure and makes it possible to understand its behavior in endotoxemia and/or sepsis.

The data obtained in our study, showing the elevation of lactate concentrations after the administration of LPS, are in accordance with the evidence in the literature^(^ 13 ^,^ 39 ^)^. This increase appears to have an immuno-modulatory effect leading to changes in thermo-regulation. However, it is necessary to expand the number of studies to explain the effect of lactate on body temperature. We believe that there is certain potential to consider body temperature assessments, associated with plasma NO and lactate concentrations, as a way to assess a change in septic patient prognosis.

The limitations of this study are related to the lack of characterization in the experimental model of septic shock, as a way to analyze the effects of endotoxemia on body temperature. The analysis of thermo-regulation in an experimental septic shock model may more clearly reflect the effects of LPS on hypothermia and plasma nitrate and lactate concentrations. Therefore, we also suggest the evaluation of these biomarkers in experimental models of septic shock.

## Conclusion

This study showed that the animals submitted to experimental sepsis showed ineffective thermo-regulation, according to the dose of LPS administered. The animals that received higher doses of LPS had a significantly lower temperature in relation to the other endotoxemic groups, which showed an increase in temperature. This behavior was accompanied by an increase in plasma NO and lactate concentrations. It was also identified that fever was correlated with high concentrations of plasma NO and lactate, important pathophysiological mediators observed during endotoxemia. The study has as its implications for Nursing the importance of monitoring body temperature, together with the assessment of these pathophysiological markers, which suggest a worsening in the prognosis of sepsis.
